# Sequential Prediction of Bronchopulmonary Dysplasia from Birth Through the First Week of Life Using Machine Learning

**DOI:** 10.3390/children13101299

**Published:** 2026-09-24

**Authors:** Younghyun Kim, Ji Eun Jeong, Sang Gyu Kwak

**Affiliations:** 1 Department of Pediatrics, Daegu Catholic University School of Medicine, Daegu 42472, Republic of Korea; wannabeyh@cu.ac.kr (Y.K.); neojeong@cu.ac.kr (J.E.J.); 2 Department of Medical Statistics, Daegu Catholic University School of Medicine, Daegu 42472, Republic of Korea

**Keywords:** bronchopulmonary dysplasia, preterm infants, machine learning, prediction model, sequential risk assessment, SHapley Additive exPlanations

## Abstract

**Highlights:**

**What are the main findings?**

• Adding first-24 h data improved BPD discrimination, with smaller additional gains from information accumulated through postnatal day 7.
• Logistic regression achieved the highest numerical test-set AUROC at each step; differences from tree-based algorithms were not statistically significant at Steps 2 or 3 after multiplicity adjustment.

**What are the implications of the main findings?**

• The findings support further evaluation of cumulative, time-aligned predictor sets for BPD risk assessment.
• Results apply to selected survivors with complete data and do not establish generalizable performance or clinical benefit.
• Temporal and external validation and prospective evaluation of clinical utility are required before implementation.

**Abstract:**

**Background/Objectives**: Bronchopulmonary dysplasia (BPD) risk evolves as clinical information accumulates after birth. This study evaluated the incremental predictive value of routinely available data from birth through the first week of life using cumulative, time-aligned feature sets. **Methods**: The primary analysis of this retrospective cohort study included 2153 preterm infants who survived to 36 weeks’ postmenstrual age and had ascertainable BPD status and complete predictor data. The outcome was clinically documented BPD at 36 weeks’ postmenstrual age, based on the treating neonatologists’ assessment of continued supplemental oxygen and/or respiratory-support requirements. Three cumulative feature sets were constructed using variables available at or immediately after birth (Step 1), additional clinical and laboratory variables available within the first 24 h (Step 2), and further clinical-course variables available through postnatal day 7 (Step 3). Logistic regression, random forest, XGBoost, LightGBM, and CatBoost models were developed using a stratified 70:30 training–test split and five-fold stratified cross-validation. Internal validation assessed discrimination, calibration, and Brier scores in the held-out test set. AUROC confidence intervals and paired differences between prediction windows were estimated using 2000 bootstrap resamples. Predictor contributions were examined using SHapley Additive exPlanations. A multiple-imputation sensitivity analysis evaluated logistic regression and random forest in all 2616 infants with ascertainable BPD status. **Results:** Of the 2153 infants, 1154 (53.6%) had clinically documented BPD. Logistic regression achieved the highest held-out test-set AUROC at each prediction window: 0.751 (95% CI, 0.714–0.788) in Step 1, 0.897 (0.873–0.919) in Step 2, and 0.923 (0.902–0.942) in Step 3. Paired AUROC increases were 0.146 (95% CI, 0.110–0.181) from Step 1 to Step 2 and 0.026 (0.015–0.036) from Step 2 to Step 3. Logistic regression Brier scores decreased from 0.202 to 0.130 and 0.112, respectively. In the Step 3 logistic regression model, the leading predictors by mean absolute SHAP value were mean SpO_2_ during the first 24 h, mean arterial pressure, initial feeding approach, FiO_2_ at 24 h, and C-reactive protein. In the expanded sensitivity-analysis test cohort, logistic-regression AUROCs were 0.747, 0.893, and 0.913 for Steps 1–3, respectively. **Conclusions**: The addition of first-24 h clinical information substantially improved BPD prediction beyond birth variables, with further improvement from data accumulated through postnatal day 7. The primary analysis did not demonstrate an added predictive benefit of the evaluated tree-based algorithms over logistic regression. The primary findings apply to a selected cohort of survivors with complete data and should not be generalized to all initially eligible infants or interpreted as prediction of BPD or death. Temporal and external validation and prospective evaluation of clinical utility are required before implementation.

## 1. Introduction

Bronchopulmonary dysplasia (BPD) remains one of the most consequential complications of prematurity, particularly among very preterm and very-low-birth-weight infants. Despite advances in neonatal respiratory care and improvements in survival, the prevention and treatment of BPD remain challenging because its development reflects a complex interaction among pulmonary immaturity, oxygen toxicity, mechanical ventilation, inflammation, infection, nutritional inadequacy, and disturbed lung development [1,2,3,4]. BPD is also associated with substantial consequences beyond the neonatal period, including persistent respiratory morbidity, impaired pulmonary function, pulmonary hypertension, neurodevelopmental impairment, and increased mortality [5,6,7,8,9,10]. Accordingly, the early identification of infants at high risk of BPD is important for risk-adapted monitoring, preventive management, parental counseling, and the design of targeted clinical trials.

The definition of BPD has evolved considerably, and differences among diagnostic criteria may affect both reported incidence and the ability of BPD status to predict later outcomes [6,7,8,9,11]. Nevertheless, BPD is clinically heterogeneous, and severity-based classifications do not fully capture subsequent pulmonary morbidity [6,8,9]. This heterogeneity, combined with the multifactorial pathogenesis of BPD, limits the usefulness of prediction approaches based on a single risk factor or a single time point. This study focused on predicting the presence or absence of clinically documented BPD at 36 weeks’ postmenstrual age and evaluating the incremental predictive value of information accumulated during the first week of life. Accordingly, BPD was treated as a binary outcome; prediction of disease severity was beyond the scope of the present study.

Numerous antenatal, perinatal, and early postnatal factors have been associated with BPD. Lower gestational age and birth weight, respiratory failure, invasive mechanical ventilation, oxygen exposure, hemodynamic instability, patent ductus arteriosus, infection, inflammation, and inadequate early nutrition have all been implicated in its development [1,2,3,4,12,13,14,15,16]. However, the availability and predictive relevance of these factors change rapidly during the first days after birth. Variables available at delivery may facilitate the earliest possible risk assessment, whereas respiratory, hemodynamic, laboratory, infectious, and nutritional information accumulated during the first week may more directly reflect the infant’s evolving clinical course. A clinically useful prediction strategy should therefore account for the temporal accumulation of information rather than treating all predictors as though they were simultaneously available.

Tree-based machine learning algorithms can accommodate nonlinear associations and interactions among predictors, providing a rationale for evaluating them alongside logistic regression. However, these capabilities do not necessarily translate into better predictive performance, and their relative value requires empirical comparison. Recent studies have applied machine learning algorithms to antenatal determinants, early neonatal variables, respiratory indices, and altitude-specific characteristics to predict the occurrence or severity of BPD [13,14,15,17,18]. Prediction of BPD at multiple postnatal time points is already established. Laughon et al. developed postnatal-age-specific models, and Greenberg et al. subsequently developed an updated NICHD estimator providing probabilities of death or BPD severity at multiple postnatal ages [19,20]. These approaches demonstrate that updating risk estimates as clinical information becomes available is not a new concept. Recent dynamic risk-stratification models have further investigated this approach [21]. The present study builds on this literature by comparing algorithms and cumulative predictor sets within a common analytical framework.

The primary analysis compared five algorithms using three cumulative predictor sets in a selected single-center cohort of preterm infants who survived to 36 weeks’ postmenstrual age and had ascertainable BPD status and complete predictor data. The predictor sets comprised information available immediately after the initial postnatal assessment, additional information available within 24 h, and further information available through postnatal day 7. The objective was to quantify changes in discrimination, calibration, and overall prediction error under a common development–test partition and to describe predictor contributions using SHAP analysis. This study provides an exploratory comparison of cumulative predictor sets and algorithms within the selected survivor cohort. It does not establish a new dynamic modeling methodology or a model for early bedside decision-making in an unselected neonatal population.

## 2. Materials and Methods

### 2.1. Study Design and Population

This retrospective cohort study screened infants admitted to the neonatal intensive care unit of Daegu Catholic University Medical Center between January 2015 and June 2025. Initial eligibility was defined as gestational age < 32 weeks or birth weight < 1500 g, without major congenital anomalies or congenital metabolic disorders. The original study criteria additionally required survival for at least 28 days after birth. However, inclusion in the primary complete-case analysis also required survival to the BPD assessment at 36 weeks’ postmenstrual age (PMA), ascertainable BPD status, and complete data for the model predictors. Thus, infants who survived 28 days but died before 36 weeks’ PMA were also excluded. The primary analytic cohort comprised 2153 infants with complete outcome and predictor data. An additional multiple-imputation sensitivity analysis included all 2616 infants with ascertainable BPD status, including 463 infants with missing predictor data.

The study was approved by the Institutional Review Board of Daegu Catholic University Medical Center (IRB No. DCUMC 2025-10-006). The requirement for informed consent was waived because of the retrospective nature of the study.

### 2.2. Outcome Definition

The primary outcome was clinically documented BPD at 36 weeks’ PMA. BPD status was obtained from the medical record and reflected the treating neonatologists’ routine clinical diagnosis based on the continued need for supplemental oxygen and/or respiratory support, including continuous positive airway pressure or mechanical ventilation, at the 36-week assessment. Clinical and radiographic findings informed routine clinical assessment but were not prespecified operational criteria for outcome classification. Specific oxygen thresholds, respiratory-support criteria, and the use of a room-air challenge could not be retrospectively verified. A uniform assessment protocol across the 2015–2025 study period could not be confirmed. The recorded clinical diagnosis was used as the binary outcome for model development and evaluation.

Infants who died before the BPD assessment at 36 weeks’ PMA were excluded from the analytic cohort and were not classified as having or not having BPD. Death before assessment was a competing outcome but was not modeled in this analysis. Among included infants, deaths occurred after the 36-week assessment, and these infants retained their BPD classification at that assessment. The models therefore estimate BPD risk conditional on survival to 36 weeks’ PMA and inclusion in the analytic cohort, rather than the risk of BPD or death in the overall initially eligible population.

### 2.3. Data Collection and Predictor Definitions

Demographic, maternal, perinatal, physiologic, respiratory, nutritional, and laboratory data were obtained from electronic medical records. Candidate predictors were selected according to their clinical relevance and their availability within three prespecified prediction windows.

Variables available at or immediately after birth included sex, airway abnormalities identified immediately after birth, hypertensive disorders of pregnancy, maternal smoking during pregnancy, gestational age, birth weight, maternal age, and 1 and 5 min Apgar scores. Because the 5 min Apgar score was included, Step 1 represented prediction immediately after the initial postnatal assessment rather than at the instant of delivery.

The additional variables assigned to the 24 h prediction window were initial feeding approach, neonatal respiratory distress syndrome, respiratory failure, endotracheal intubation, peripheral oxygen saturation (SpO_2_), fraction of inspired oxygen (FiO_2_), heart rate, mean arterial pressure, body temperature, hemoglobin, hematocrit, white blood cell count, platelet count, C-reactive protein, aspartate aminotransferase, alanine aminotransferase, and total parenteral nutrition use. Initial feeding approach was classified within the first 24 h after birth as breast milk or formula. Infants in whom breast-milk feeding was initiated were assigned to the breast-milk category. When breast-milk feeding could not be initiated, formula feeding was attempted and the infant was assigned to the formula category. This variable therefore captured the initial feeding approach rather than confirmed milk administration; classification in the formula category did not necessarily indicate successful formula feeding. For model fitting, initial feeding approach was dummy-coded as formula = 1 and breast milk = 0, with breast milk serving as the reference category. SpO_2_, heart rate, mean arterial pressure, and temperature were summarized as means over the first 24 h. FiO_2_ represented the value recorded at 24 h. For each laboratory predictor, the first result available within 24 h after birth was used. Eligibility for inclusion was based on result availability within this window, rather than specimen collection time alone.

Additional variables assigned to the 7-day prediction window included any invasive mechanical ventilation during the first 7 days, mean FiO_2_ during the first week, early-onset infection identified within 72 h after birth, late-onset infection identified after 72 h and through postnatal day 7, coagulation abnormalities, and thrombocytopenia. Invasive mechanical ventilation within 7 days was coded as a binary indicator of any exposure during this interval. Mean FiO_2_ during the first week was calculated by first obtaining the mean FiO_2_ for each of the first seven postnatal days and then taking the arithmetic mean of these seven daily means, with equal weight assigned to each day.

### 2.4. Construction of Three Hierarchical Feature Sets

Three cumulative feature sets were constructed to evaluate the additional predictive information obtained as clinical data accumulated. Step 1 included nine predictors available at or immediately after birth. Step 2 added 17 clinical, physiologic, laboratory, and nutritional predictors assigned to the first 24 h, yielding 26 predictors. Step 3 added six clinical-course predictors assigned to the first 7 days, yielding 32 predictors. Patent ductus arteriosus (PDA) and final blood-culture results were excluded from all three feature sets, and initial feeding approach was included only in Steps 2 and 3.

The feature sets were cumulative rather than independent. Accordingly, differences in performance between consecutive steps represented the additional predictive information available at later clinical time points. Step 1, Step 2, and Step 3 therefore represented prediction immediately after the initial postnatal assessment, at 24 h, and at 7 days, respectively.

### 2.5. Data Preprocessing and Dataset Partitioning

The primary analysis included only infants with complete outcome and predictor data; no missing-value imputation was performed for this analysis. To describe selection associated with missing predictors, available baseline characteristics and observed BPD status were compared between infants excluded from the primary analysis and those included. Comparisons used variable-specific available sample sizes. Continuous variables were compared using Welch’s *t*-test and binary categorical variables using the chi-square test with continuity correction. Absolute standardized mean differences were calculated using the unweighted average of the two group variances. This descriptive comparison did not correct for selection bias. A separate multiple-imputation sensitivity analysis was performed as described in Section 2.9.

The primary complete-case–cohort was randomly divided into development and held-out test sets at a 70:30 ratio using outcome-stratified sampling (random_state = 42). The development set comprised 1507 infants, including 808 with BPD and 699 without BPD; the test set comprised 646 infants, including 346 with BPD and 300 without BPD. Five-fold stratified cross-validation was performed within the development set. The same infant-level development–test partition was used for all feature sets and algorithms. This procedure constituted internal validation within the selected single-center cohort. Under the institutional data-review and privacy-protection procedures overseen by the hospital’s Data Review Committee, dates of birth and admission and maternal identifiers were withheld from the Electronic medical record (EMR) derived research dataset as personal information. Patient-level calendar-year information and a pregnancy linkage identifier were also unavailable in the analytic extract. Consequently, a chronological split could not be implemented, and neither the development–test partition nor the cross-validation folds could be grouped by pregnancy. No temporal or external validation was performed.

For logistic regression, all numeric predictors, including binary variables stored numerically, were standardized using means and standard deviations estimated exclusively from the applicable training data and then applied unchanged to the corresponding validation or test data. Categorical predictors were one-hot encoded, with one reference category omitted for binary categorical variables, and were not standardized. Tree-based models were fitted without standardization. All preprocessing parameters were estimated using only the applicable training data.

### 2.6. Model Development and Evaluation

In the primary analysis, five classification algorithms were evaluated: logistic regression, random forest, extreme gradient boosting (XGBoost), Light Gradient Boosting Machine (LightGBM), and CatBoost. Hyperparameters were prespecified and applied consistently across the three feature sets without data-driven feature selection or feature-set-specific optimization. Complete model specifications are provided in Supplementary Table S1.

Within the training set, model performance was assessed using five-fold stratified cross-validation with shuffling (random_state = 42). In each fold, preprocessing parameters were estimated using only the four training folds and then applied to the remaining validation fold. Cross-validation performance was summarized as the mean and standard deviation across the five validation folds.

After cross-validation, each model was refitted using the complete training set and evaluated once in the held-out test set. Model performance was evaluated using accuracy and the area under the receiver operating characteristic curve (AUROC). Predicted probabilities were converted to binary classifications using a fixed threshold of 0.5 solely to provide a common basis for comparing accuracy across models. This threshold was not selected according to clinical consequences and should not be interpreted as a clinically recommended cutoff. AUROC, calibration, and Brier scores were evaluated using predicted probabilities. Calibration was assessed graphically by comparing predicted and observed BPD probabilities across 10 groups defined by quantiles of predicted risk, with shaded areas representing 95% confidence intervals.

### 2.7. Model Interpretation Methods

Global model interpretation was performed using conventional feature-importance measures and SHapley Additive exPlanations (SHAP). For logistic regression, predictor importance was represented by the absolute magnitude of the regression coefficients after preprocessing. Model-specific importance measures were used for the tree-based algorithms.

Mean absolute SHAP values, denoted as mean(|SHAP|), were calculated to quantify the overall contribution of each predictor to model output. SHAP summary plots were used to examine the distributions of predictor values and their corresponding contributions across individuals. These analyses describe the behavior of the fitted prediction models and should not be interpreted as estimates of causal effects.

### 2.8. Statistical Analysis

Continuous variables are presented as means with standard deviations, and categorical variables as frequencies with percentages. Baseline and clinical characteristics were compared descriptively between infants with and without BPD. Continuous variables were compared using Welch’s independent-samples *t*-test. Categorical variables were compared using the chi-square test or Fisher’s exact test, as appropriate. These comparisons were conducted for descriptive purposes and were not used to screen or select model predictors. All statistical tests were two-sided, and a *p*-value < 0.05 was considered statistically significant. Analyses were performed using Python version 3.11 with scikit-learn version 1.8.0, XGBoost version 3.0.5, LightGBM version 4.6.0, CatBoost version 1.2.8, and SHAP version 0.52.0.

Test-set AUROC and Brier score uncertainty was quantified using 2000 nonparametric bootstrap resamples of individual infants, with 95% percentile confidence intervals. Incremental discrimination between consecutive prediction windows was assessed using paired bootstrap resampling: the same infant indices were used for both models in each resample, and the difference in AUROC was calculated as the later-window estimate minus the earlier-window estimate. These revision-stage comparisons were exploratory, and confidence intervals were not adjusted for multiplicity. Models were held fixed during resampling; thus, the intervals reflect test-sample uncertainty conditional on the fitted models and data partition.

Exploratory comparisons between algorithms were performed using two-sided paired DeLong tests, comparing logistic regression with each of the four tree-based algorithms within each prediction window. Holm adjustment was applied across the 12 comparisons. AUROC differences are reported as logistic regression minus the comparator, with nominal 95% DeLong confidence intervals that were not adjusted for multiplicity. These comparisons were conditional on the fitted models and the selected test cohort.

Calibration-in-the-large was estimated as the intercept of a logistic model with the logit of predicted probability included as an offset and its coefficient fixed at 1. Calibration slope was estimated using a separate logistic model with a freely estimated intercept. Model-based Wald 95% confidence intervals were calculated for these calibration parameters. Calibration models were used for evaluation only, without recalibrating the test predictions. Overall prediction error was assessed using the Brier score. Exploratory decision-curve analysis was performed using unchanged held-out test predictions for all five algorithms at each prediction window. Net benefit was calculated as TP/N − (FP/N) × pt/(1 − pt), where TP and FP denote true-positive and false-positive counts, N is the test-set size, and pt is the threshold probability. Thresholds from 0.05 to 0.80 in increments of 0.01 were examined and compared with hypothetical treat-all and treat-none strategies. This range was selected for exploratory evaluation; no specific intervention, clinically justified threshold range, or optimal cutoff was established [22].

### 2.9. Methods for Multiple-Imputation Sensitivity Analysis

A sensitivity analysis included all 2616 infants with ascertainable BPD status. The original allocation of 1507 development and 646 test infants was retained. The 463 previously excluded infants were independently divided using outcome-stratified 70:30 sampling (random_state = 42), adding 324 development and 139 test infants. The expanded datasets therefore comprised 1831 development and 785 test infants.

For each prediction window, 20 independent imputations were generated using 20 iterations of chained predictive mean matching with five donors, type-1 matching, Bayesian linear-regression coefficient draws, and a ridge constant of 10^−5^. Only predictors assigned to the corresponding prediction window were used. Conditional imputation models and observed donor pools were estimated exclusively from development infants and used to impute missing predictors in both partitions.

Logistic regression and random forest were refitted in each imputed development dataset using the original fixed hyperparameters. Predicted probabilities for each test infant were averaged across the 20 imputations. Discrimination, calibration, and Brier scores were evaluated in the expanded test cohort and separately in the original complete-case and previously excluded test subsets. Paired AUROC changes were also evaluated in the original 646 test infants. AUROC confidence intervals were obtained using 2000 bootstrap resamples of test infants, with fitted models and averaged predictions held fixed.

This sensitivity analysis used a custom Python implementation of chained predictive mean matching with Python 3.12.14, NumPy 2.3.5, pandas 2.2.3, SciPy 1.17.0, and scikit-learn 1.8.0.

## 3. Results

### 3.1. Study Population and Birth Characteristics

Of the 5153 infants initially screened, 2085 did not meet the gestational-age and/or birth-weight criteria, 173 had major congenital anomalies, and 22 had congenital metabolic disorders, leaving 2873 infants meeting the initial eligibility criteria (Figure 1). BPD status at 36 weeks’ PMA was unavailable for 257 infants: 10 were discharged without subsequent outcome ascertainment, 60 were transferred without subsequent outcome ascertainment, 180 died before 36 weeks’ PMA, and seven had no documented assessment despite continued hospitalization or follow-up. Among the 180 infants who died, 155 died before 28 days of age and 25 died at or after 28 days but before 36 weeks’ PMA. Of the remaining 2616 infants with ascertainable BPD status, 463 were excluded from the primary complete-case analysis because of missing predictor data. The primary analytic cohort comprised 2153 infants, including 999 (46.4%) without BPD and 1154 (53.6%) with BPD. All 2616 infants with ascertainable BPD status were included in the additional multiple-imputation sensitivity analysis.

Supplementary Table S2 compares infants excluded because of missing predictor data with those included in the primary analysis. The observed BPD proportions were 54.6% and 53.6%, respectively (absolute SMD, 0.021). Excluded infants had a lower mean gestational age (27.34 versus 28.82 weeks; absolute SMD, 0.448). Differences were also observed in birth weight, maternal age, and 1 and 5 min Apgar scores, with absolute SMDs ranging from 0.136 to 0.221. These differences indicate that the complete-case–cohort differed from infants excluded because of missing predictor data.

Birth and perinatal characteristics are summarized in Table 1. Compared with infants without BPD, those with BPD had a lower mean gestational age (28.30 ± 3.41 vs. 29.41 ± 3.41 weeks; *p* < 0.001), lower mean birth weight (1152.36 ± 360.99 vs. 1310.04 ± 411.82 g; *p* < 0.001), and lower Apgar scores at both 1 and 5 min (both *p* < 0.001). Airway malformations were more frequent among infants with BPD (3.3% vs. 1.5%; *p* = 0.011), as were hypertensive disorders of pregnancy (13.2% vs. 8.4%; *p* < 0.001) and maternal smoking during pregnancy (12.4% vs. 7.7%; *p* < 0.001). Maternal age was also higher in the BPD group (28.62 ± 6.01 vs. 27.52 ± 5.75 years; *p* < 0.001). The proportion of male infants did not differ significantly between the groups (55.6% vs. 53.3%; *p* = 0.288).

### 3.2. Clinical and Laboratory Characteristics Within the First 24 H

Clinical and laboratory variables available within the first 24 h after birth are presented in Table 2. Neonatal respiratory distress syndrome was more frequent in the BPD group than in the non-BPD group (74.9% vs. 61.6%; *p* < 0.001), as were respiratory failure (59.7% vs. 43.3%; *p* < 0.001) and endotracheal intubation (27.7% vs. 11.8%; *p* < 0.001). Infants with BPD were more frequently classified in the formula category of the initial feeding approach (56.8% vs. 38.0%; *p* < 0.001). This category included attempted formula feeding when breast-milk feeding could not be initiated and did not necessarily indicate actual formula administration.

Infants with BPD had lower mean SpO_2_, mean arterial pressure, and mean temperature during the first 24 h, together with higher mean heart rate and FiO_2_ at 24 h (all *p* < 0.001). Based on the first laboratory results available within 24 h, the BPD group had lower hemoglobin, hematocrit, and platelet count and higher white blood cell count, C-reactive protein, aspartate aminotransferase, and alanine aminotransferase levels (all *p* < 0.001). Total parenteral nutrition use within 24 h was also more frequent in the BPD group (60.0% vs. 53.6%; *p* = 0.003).

### 3.3. Clinical Course During the First 7 Days

Clinical-course variables observed through the end of postnatal day 7 are summarized in Table 3. Infants in the BPD group more frequently received invasive mechanical ventilation during the first 7 days than those in the non-BPD group (32.1% vs. 17.7%; *p* < 0.001). Mean FiO_2_ during the first week, calculated as the arithmetic mean of the seven daily means, was also higher in the BPD group (0.30 ± 0.10 vs. 0.25 ± 0.09; *p* < 0.001).

Late-onset infection identified after 72 h and through the end of postnatal day 7 was more frequent in the BPD group (26.4% vs. 21.4%; *p* = 0.008). Coagulation abnormalities (34.9% vs. 18.1%; *p* < 0.001) and thrombocytopenia (13.2% vs. 4.4%; *p* < 0.001) were also more common in the BPD group. The frequency of early-onset infection did not differ significantly between the groups (4.4% vs. 2.9%; *p* = 0.082).

### 3.4. Outcomes During Hospitalization

Clinical characteristics and outcomes recorded during hospitalization are presented in Table 4. Compared with infants without BPD, those with BPD had higher frequencies of PDA diagnosed by echocardiography after 24 h (51.0% vs. 40.3%; *p* < 0.001) and final blood-culture positivity (11.7% vs. 4.4%; *p* < 0.001). Invasive mechanical ventilation, high-frequency ventilation, transfusion, intravenous glucocorticoid therapy, and caffeine therapy were also more frequent in the BPD group (all *p* < 0.001). Infants with BPD had longer durations of invasive mechanical ventilation (17.89 ± 10.34 vs. 11.22 ± 8.23 days), supplemental oxygen therapy (37.62 ± 18.34 vs. 26.23 ± 17.33 days), antibiotic use (7.76 ± 3.64 vs. 6.13 ± 3.17 days), and nutritional support (25.19 ± 9.78 vs. 15.31 ± 7.94 days), as well as a longer hospital stay (68.23 ± 25.05 vs. 50.46 ± 24.48 days; all *p* < 0.001).

Among infants included in the analytic cohort, 150 died during hospitalization after BPD status had been assessed at 36 weeks’ PMA. All these deaths occurred in the BPD group (150/1154 [13.0%]), whereas none occurred in the non-BPD group (0/999 [0.0%]; *p* < 0.001).

The variables in Table 4 were summarized for descriptive purposes. PDA, final blood-culture results, and measures covering the entire hospitalization were not used as predictors. The separate binary indicator of invasive mechanical ventilation within the first 7 days was included in Step 3.

### 3.5. Model Performance Across Prediction Windows

Model performance across the three cumulative feature sets is summarized in Table 5. Discrimination improved as information accumulated from birth through postnatal day 7. For Step 1, mean cross-validated AUROCs ranged from 0.672 for LightGBM to 0.734 for logistic regression. Held-out test AUROCs ranged from 0.663 for LightGBM to 0.751 for logistic regression. Logistic regression also achieved the highest test accuracy of 0.687. For Step 2, mean cross-validated AUROCs ranged from 0.916 for random forest to 0.924 for CatBoost. Test AUROCs ranged from 0.892 to 0.897, with logistic regression achieving the highest value. XGBoost and LightGBM achieved the highest test accuracy of 0.808. For Step 3, mean cross-validated AUROCs ranged from 0.926 for random forest to 0.937 for CatBoost. Test AUROCs ranged from 0.909 for LightGBM to 0.923 for logistic regression. Logistic regression achieved the highest test accuracy of 0.839. ROC curves in Figure 2 show the same pattern. The largest increase in discrimination occurred between Steps 1 and 2, with a smaller additional increase between Steps 2 and 3. Pairwise comparisons between algorithms are presented in Supplementary Table S3. After Holm adjustment, logistic regression had higher AUROC than XGBoost, LightGBM, and CatBoost at Step 1 (adjusted *p* < 0.001, <0.001, and 0.008, respectively), but the difference from random forest was not significant (adjusted *p* = 0.088). No significant differences between logistic regression and the tree-based algorithms were found at Steps 2 or 3. At Step 3, the AUROC difference between logistic regression and XGBoost was 0.0123 (95% CI, −0.0001 to 0.0247; unadjusted *p* = 0.052; adjusted *p* = 0.414).

The logistic regression test AUROCs were 0.751 (95% CI, 0.714–0.788), 0.897 (0.873–0.919), and 0.923 (0.902–0.942) for Steps 1, 2, and 3, respectively. Paired bootstrap comparisons showed an AUROC increase of 0.146 (95% CI, 0.110–0.181) from Step 1 to Step 2 and 0.026 (0.015–0.036) from Step 2 to Step 3. Both intervals excluded zero, indicating incremental discrimination within the selected test cohort. The gray dashed diagonal line represents chance-level discrimination (AUROC = 0.5). Comparisons for all five algorithms are provided in Supplementary Table S4.

### 3.6. Calibration and Exploratory Decision-Curve Analysis

Calibration plots are presented in Figure 3, and quantitative calibration measures and Brier scores are reported in Supplementary Table S5. For logistic regression, calibration-in-the-large estimates were 0.058, 0.109, and 0.009, and calibration slopes were 1.085, 0.890, and 0.948 for Steps 1, 2, and 3, respectively. The Step 3 estimates were 0.009 (95% CI, −0.227 to 0.246) for calibration-in-the-large and 0.948 (0.804–1.092) for calibration slope.

Logistic regression Brier scores decreased from 0.202 (95% CI, 0.190–0.215) in Step 1 to 0.130 (0.114–0.147) in Step 2 and 0.112 (0.097–0.128) in Step 3. Calibration varied across algorithms despite similar discrimination at the later prediction windows. In Step 3, calibration slopes ranged from 0.354 for LightGBM to 2.262 for random forest, indicating that similar AUROCs did not imply comparable calibration.

Exploratory decision curves are presented in Supplementary Figure S1. The Step 2 and Step 3 logistic regression models had higher estimated net benefit than both default strategies throughout the evaluated threshold grid of 0.05–0.80. At an illustrative threshold of 0.20, net benefit was 0.423, 0.457, and 0.466 for the Step 1, Step 2, and Step 3 logistic regression models, respectively, compared with 0.420 for treat all and 0 for treat none. This example does not imply that 0.20 is a recommended clinical cutoff.

### 3.7. Model Interpretation

Absolute regression coefficients and mean absolute SHAP values for the logistic regression models across all three prediction windows are provided in Supplementary Table S6. SHAP plots for the Step 1 and Step 2 logistic regression models are presented in Supplementary Figures S2 and S3, respectively. Figure 4 presents SHAP-based interpretation of the Step 3 logistic regression model. Ranked by mean absolute SHAP value, the 10 leading predictors were mean SpO_2_ during the first 24 h, mean arterial pressure, initial feeding approach, FiO_2_ at 24 h, C-reactive protein, the 5 min Apgar score, hematocrit, coagulation abnormalities, white blood cell count, and intubation. Lower SpO_2_, mean arterial pressure, 5 min Apgar scores, and hematocrit generally contributed to higher model output for BPD. Higher FiO_2_, C-reactive protein, and white blood cell count, together with assignment to the formula category of the initial feeding approach, coagulation abnormalities, and intubation, generally contributed to higher model output for BPD. These findings describe associations within the fitted prediction model and should not be interpreted as causal effects. Adjusted odds ratios with 95% confidence intervals from the supplementary multivariable logistic regression analysis using the Step 3 predictor set in the full analytic cohort are presented in Supplementary Table S7.

### 3.8. Multiple-Imputation Sensitivity Analysis

The sensitivity analysis included 2616 infants, of whom 1407 (53.8%) had BPD. In the expanded test cohort of 785 infants, logistic-regression AUROCs were 0.747 (95% CI, 0.715–0.780), 0.893 (0.870–0.913), and 0.913 (0.892–0.931) for Steps 1–3, respectively. Corresponding Brier scores were 0.204, 0.134, and 0.120. Random-forest AUROCs were 0.729, 0.893, and 0.902, respectively. For the Step 3 logistic-regression model, calibration-in-the-large was 0.006 and the calibration slope was 0.937 (Supplementary Table S8).

When the refitted logistic-regression models were evaluated in the same original 646 complete-case test infants, AUROCs were 0.753, 0.898, and 0.922 for Steps 1–3, respectively. The paired AUROC changes from the original models were +0.0014 (95% CI, −0.0051 to +0.0082), +0.0004 (−0.0026 to +0.0033), and −0.0012 (−0.0051 to +0.0029). Among the 139 previously excluded test infants, the Step 3 logistic-regression AUROC was 0.873 (95% CI, 0.809–0.926), with calibration-in-the-large of −0.437 and a calibration slope of 0.875. Predictor-specific missingness before imputation is reported in Supplementary Table S9.

## 4. Discussion

In this selected retrospective cohort of 2153 preterm infants who survived to 36 weeks’ PMA and had ascertainable BPD status and complete predictor data, BPD prediction improved progressively as clinical information accumulated during the first week after birth. Models based solely on variables available at birth showed moderate discrimination, whereas adding respiratory, physiologic, and laboratory data from the first 24 h substantially improved performance. Information obtained through postnatal day 7 provided a further, although smaller, improvement. The highest test-set AUROC increased from 0.751 in Step 1 to 0.897 in Step 2 and 0.923 in Step 3. These findings quantify the incremental predictive information provided by cumulative, time-aligned feature sets within this selected survivor cohort.

The largest performance gain occurred after incorporating information from the first 24 h. Birth characteristics such as gestational age, birth weight, and Apgar scores reflect baseline vulnerability but do not fully capture the early respiratory and systemic disturbances involved in BPD development. In contrast, oxygen requirement, oxygen saturation, respiratory failure, hemodynamic measurements, inflammatory markers, and hematologic indices reflect the infant’s initial adaptation and severity of illness. The additional improvement in Step 3 suggests that the subsequent first-week course—including mechanical ventilation, oxygen exposure, infection, coagulation abnormalities, and thrombocytopenia—further refines risk estimation. Prediction of BPD at multiple postnatal ages is well established. Laughon et al. developed postnatal-age-specific models incorporating BPD severity and the competing outcome of death in a multicenter cohort [19]. Greenberg et al. subsequently updated the NICHD estimator to predict BPD grades or death using contemporary data and respiratory-support information at multiple postnatal ages [20]. More recently, Zhao et al. developed severity-stratification models using information from postnatal days 1, 3, and 7 and evaluated selected models in a prospective external cohort from six neonatal centers [21].

The present study complements these approaches through a common comparative framework. All five algorithms and three cumulative predictor sets were evaluated in the same analytic cohort using identical development–test partitions. Retaining earlier predictors while adding information available at later windows allowed paired quantification of incremental discrimination, alongside assessment of calibration and overall prediction error. This design reduces differences attributable to cohort composition or data partitioning when comparing prediction windows, although it does not remove selection bias or establish a new dynamic modeling methodology.

Important limitations remain relative to previous approaches. The primary models predict binary clinically documented BPD among survivors with complete data, without estimating disease severity or the competing outcome of death. Evaluation was limited to internal validation at a single center, and no direct comparison with an established BPD estimator was performed. Differences in eligibility criteria, outcome definitions, prediction times, and validation designs preclude interpreting cross-study AUROC differences as evidence of superiority. The findings therefore quantify the additional information available during the first week within this selected cohort, rather than establish a validated system for updating risk in all infants remaining at risk at each postnatal age.

In the primary analysis, the increase in AUROC from Step 1 to Step 2 was larger than the differences among algorithms within any prediction window, whereas the additional gain from Step 2 to Step 3 was smaller. Logistic regression achieved the highest numerical test-set AUROC at all three steps and the highest test accuracy at Steps 1 and 3; XGBoost and LightGBM achieved the highest accuracy at Step 2. Differences in AUROC between logistic regression and the tree-based algorithms were not statistically significant at Steps 2 or 3 after multiplicity adjustment. Thus, the observed improvement across prediction windows reflected the additional information available in the cumulative predictor sets, without demonstrating an added benefit from the evaluated tree-based algorithms. Under the prespecified model settings and internal validation design, these results do not support preferential use of the more complex algorithms over logistic regression. However, the absence of statistically significant differences does not establish equivalence, and these findings should not be generalized to other populations or modeling strategies. SHAP analysis identified oxygen saturation, mean arterial pressure, initial feeding approach, C-reactive protein, Apgar scores, oxygen requirements, and hematologic variables as influential predictors. These variables collectively reflect respiratory compromise, inflammation, hemodynamic instability, and hematologic dysfunction, consistent with the multifactorial pathogenesis of BPD [2,3,4,16]. However, SHAP values explain model predictions rather than causal effects, and they should not be interpreted as evidence that modifying an individual predictor would alter BPD risk. Specifically, the contribution of the initial feeding approach should not be interpreted as an effect of formula exposure, because this category reflected the feeding approach and did not necessarily indicate actual formula administration.

These findings provide a basis for further research on BPD risk assessment at different postnatal times. The present analysis does not establish that using these models for risk stratification, treatment decisions, or clinical-trial enrollment would improve outcomes or study efficiency. Before such applications are considered, temporal and external validation and prospective evaluation of the intended decisions, relevant risk thresholds, and potential benefits and harms are required.

This study has several strengths, including assessment of BPD status at a common postmenstrual age, a common analytical framework across five algorithms, and cumulative feature sets aligned with clinically relevant prediction times. However, the retrospective single-center design limits generalizability. Selection of the analytic cohort is an important limitation. Infants who died before 36 weeks’ PMA or lacked an ascertainable BPD outcome were excluded from both analyses, whereas infants with missing predictor data were excluded from the primary complete-case analysis but included in the multiple-imputation sensitivity analysis. The models therefore describe BPD risk among selected survivors with available data, rather than among all infants meeting the initial gestational-age or birth-weight criteria. Survival to 36 weeks’ PMA is not known at the intended prediction times, which limits direct application of these models to an unselected population at birth, 24 h, or 7 days. Death before BPD assessment is a competing outcome that was not modeled. Moreover, the differences between excluded and included infants in Supplementary Table S2 indicate that complete-case selection may have affected predictor–outcome relationships and performance estimates. Similar observed BPD proportions do not exclude selection bias. The multiple-imputation sensitivity analysis extended evaluation to infants with incomplete predictors, and changes in logistic-regression AUROC were small when assessed in the same original test infants. However, these findings do not establish the absence of selection bias. The original and expanded test cohorts differ in case mix, so their AUROC difference cannot be interpreted directly as the amount of optimism attributable to complete-case selection. The results also depend on the imputation specification and assumptions about missingness. Outcome-blind imputation may attenuate predictor–outcome associations, and missing-not-at-random mechanisms were not examined. The sensitivity analysis was limited to logistic regression and random forest; the other algorithms, regression-coefficient inference, and SHAP interpretations were not reassessed. These analyses do not resolve selection associated with survival to 36 weeks’ PMA or replace evaluation in broader cohorts with temporal and external validation. The reduction in performance from cross-validation to the held-out test set may reflect some overfitting and optimism in development-stage estimates, as well as sampling variability. Accordingly, interpretation should prioritize the held-out test-set estimates and their uncertainty. However, this internal evaluation does not establish performance in other clinical settings or periods.

Outcome ascertainment is another limitation. BPD status was based on routine clinical diagnosis at 36 weeks’ PMA, and the available outcome variable does not establish that uniform oxygen thresholds and respiratory-support criteria were applied throughout 2015–2025. Changes in oxygen prescribing, respiratory-support practices, and diagnostic documentation may have introduced variation in outcome classification. The direction and magnitude of any resulting misclassification cannot be determined from the present analysis. Internal validation using the same clinical outcome labels does not resolve this concern. Further validation should use an explicit, standardized outcome definition applied consistently across patients and study periods.

The validation strategy provides only internal evidence of model performance. Random splitting across 2015–2025 allowed infants from overlapping clinical eras to enter both development and test datasets and did not assess performance in a later period. Changes in neonatal care and outcome ascertainment may therefore limit temporal transportability. Temporal validation could not be undertaken because patient-level calendar information was unavailable in the analytic dataset. Maternal and pregnancy identifiers were also unavailable, so siblings could not be assigned to the same partition or cross-validation fold. Shared maternal and perinatal characteristics may have introduced dependence across partitions and potentially optimistic performance estimates, although the extent of this effect could not be quantified. Estimating preprocessing parameters exclusively from training data limited information leakage through preprocessing but did not address temporal transportability or potential sibling dependence. Neither the internal resampling procedures nor the multiple-imputation sensitivity analysis resolves these limitations, and their effects on the reported performance estimates could not be quantified. No external validation was performed. The reported test-set AUROCs therefore should not be interpreted as evidence of generalizable clinical performance across periods or institutions.

Although the paired comparisons indicated incremental discrimination between prediction windows, statistical improvement does not establish clinical benefit. In particular, the additional discrimination obtained using information through day 7 does not demonstrate that delaying a decision until that time would improve outcomes. The fixed classification threshold of 0.5 was used only for model comparison and does not represent a clinically recommended cutoff. Exploratory decision-curve analysis examined net benefit across varying thresholds, but no specific model-guided intervention or clinically justified threshold range was established. The curves therefore do not demonstrate improved patient outcomes or justify delaying decisions until a later prediction window. Clinical thresholds require evaluation of the intended intervention, the consequences of false-positive and false-negative decisions, and performance in an appropriate target population. The confidence intervals quantify uncertainty within the selected test cohort conditional on the fitted models; they do not capture variability from the entire model-development process or potential dependence among siblings. Prospective evaluation of model-guided decisions and their benefits and harms remains necessary. Several predictors, including initial feeding approach, FiO_2_, and intubation, reflect clinical management as well as illness severity. The formula category represented the initial feeding approach and did not necessarily indicate actual formula administration. The analysis did not separate the contributions of underlying illness, treatment selection, and possible treatment effects. Consequently, SHAP values should not be interpreted as causal effects, biological importance, or evidence supporting changes in feeding or respiratory management. Differences in feeding policies, oxygen targets, and thresholds for intubation across institutions may alter model performance and the magnitude and ranking of predictor contributions. The reported SHAP findings therefore require external evaluation and should not be assumed to generalize to settings with different clinical practices. The binary outcome does not capture clinically important differences in severity and long-term prognosis among infants with BPD. These models therefore cannot distinguish mild, moderate, and severe BPD or provide severity-specific prognostic estimates. Future studies should evaluate prediction of BPD severity and longer-term respiratory and neurodevelopmental outcomes.

## 5. Conclusions

In the primary analysis of this selected single-center cohort of infants who survived to 36 weeks’ PMA and had ascertainable BPD status and complete predictor data, adding first-24 h information improved discrimination, with a smaller additional improvement after incorporating information through postnatal day 7. Logistic regression also showed progressively lower Brier scores across these prediction windows. The primary analysis did not demonstrate an added predictive benefit of the evaluated tree-based algorithms over logistic regression, and greater algorithmic complexity was not supported by the present results. These findings provide an exploratory comparison of cumulative predictor sets and algorithms for clinically documented BPD. They do not establish generalizable performance, superiority over existing BPD prediction tools, or clinical utility. Survivor and complete-case selection, uncertainty in outcome standardization, and the limitations of internal validation constrain interpretation. Further evaluation in broader cohorts, with standardized outcomes, temporal and external validation, and assessment of model-guided clinical decisions, is required before clinical application.

## Figures and Tables

**Figure 1:**
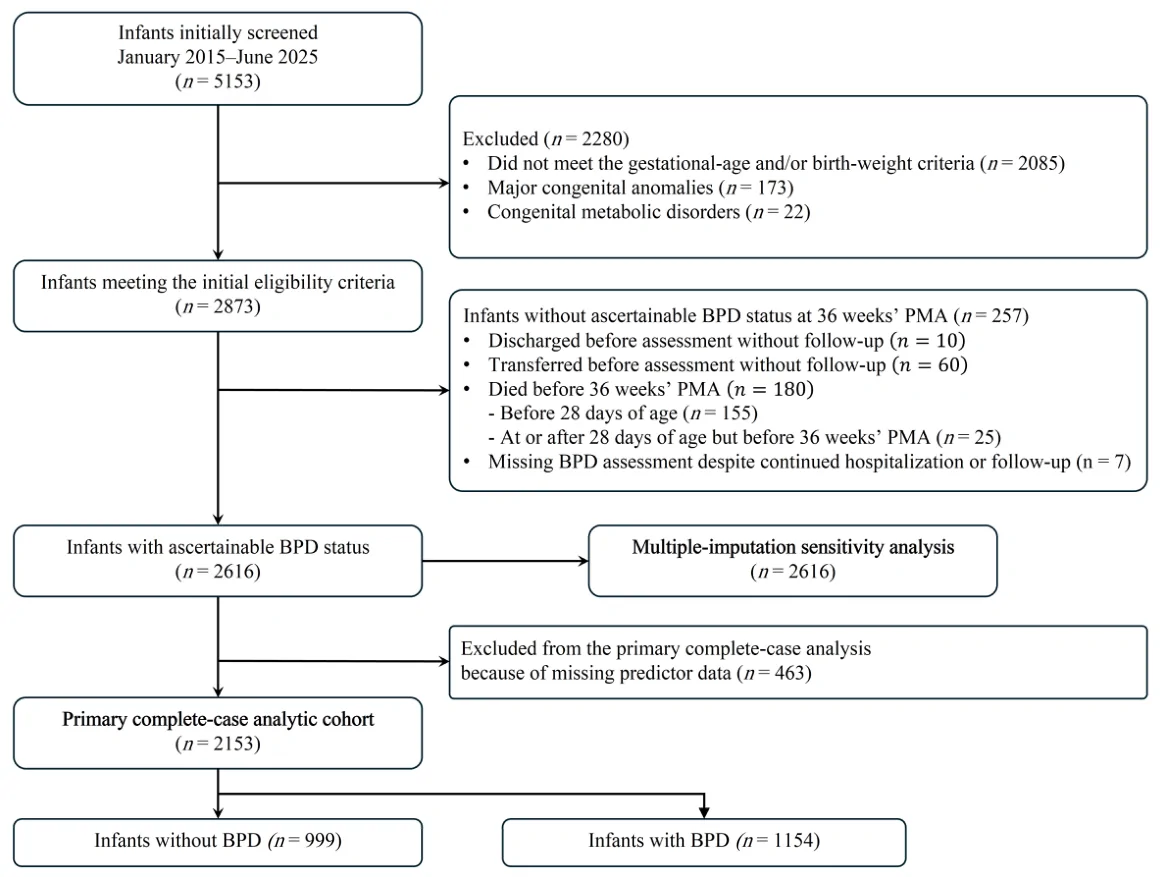
Flowchart of study participant selection. Of 5153 infants initially screened, 2280 were excluded by the eligibility criteria. Among 2873 initially eligible infants, 257 had unavailable BPD status at 36 weeks’ PMA. Of the remaining 2616 infants with ascertainable BPD status, 463 had missing predictor data and were excluded from the primary complete-case analysis, leaving 2153 infants. All 2616 infants with ascertainable BPD status were included in the additional multiple-imputation sensitivity analysis.

**Figure 2:**
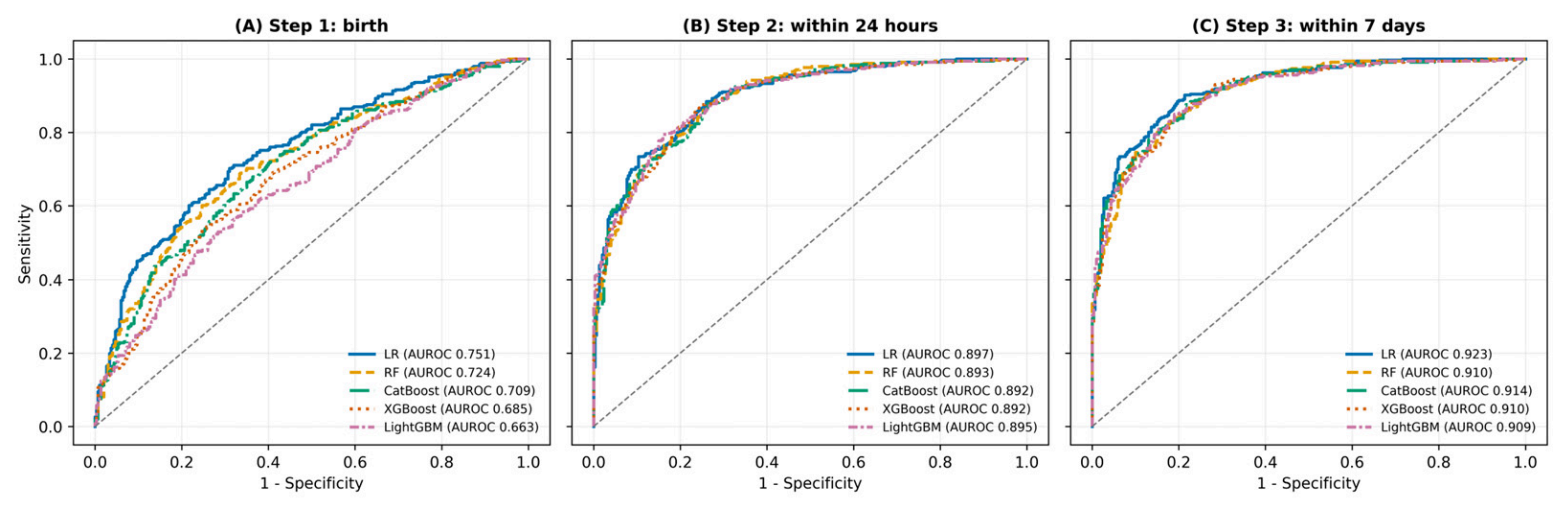
Receiver operating characteristic curves for the five prediction models across the three cumulative prediction windows. Step 1 included nine predictors available at birth; Step 2 added clinical, physiologic, laboratory, and nutrition information available within the first 24 h; and Step 3 added clinical-course variables available through postnatal day 7. PDA and final blood-culture results were excluded from all prediction models.

**Figure 3:**
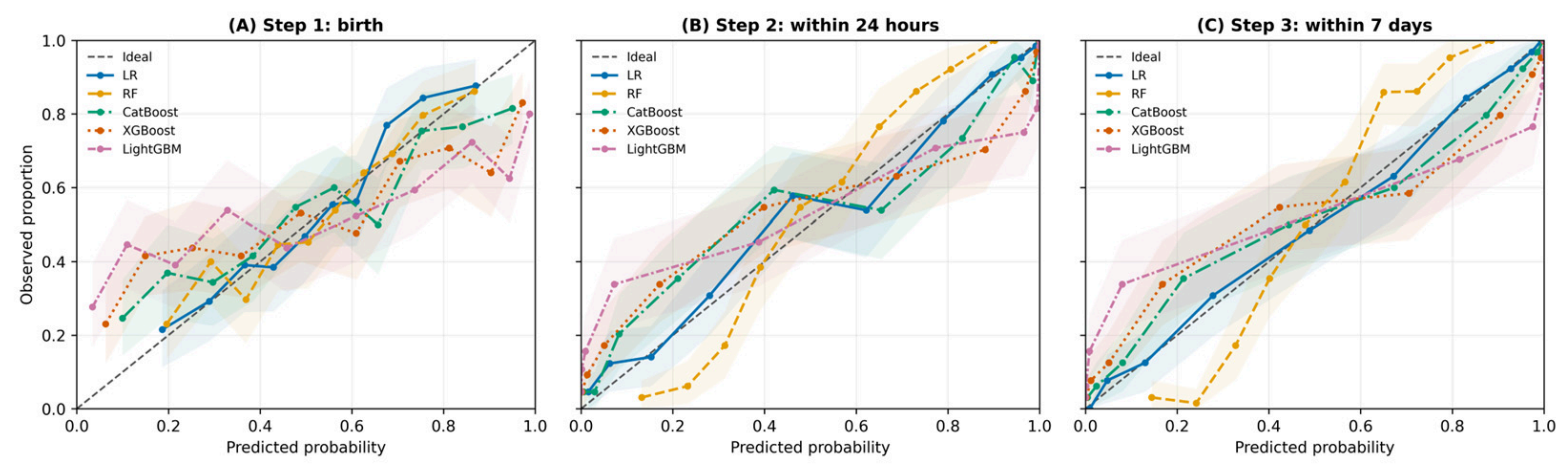
Calibration plots for the five prediction models in the held-out test set. Points show observed BPD proportions in 10 fixed quantile-based groups of predicted risk; shaded bands show nonparametric bootstrap 95% confidence intervals using the original bin boundaries. The diagonal line represents ideal calibration.

**Figure 4:**
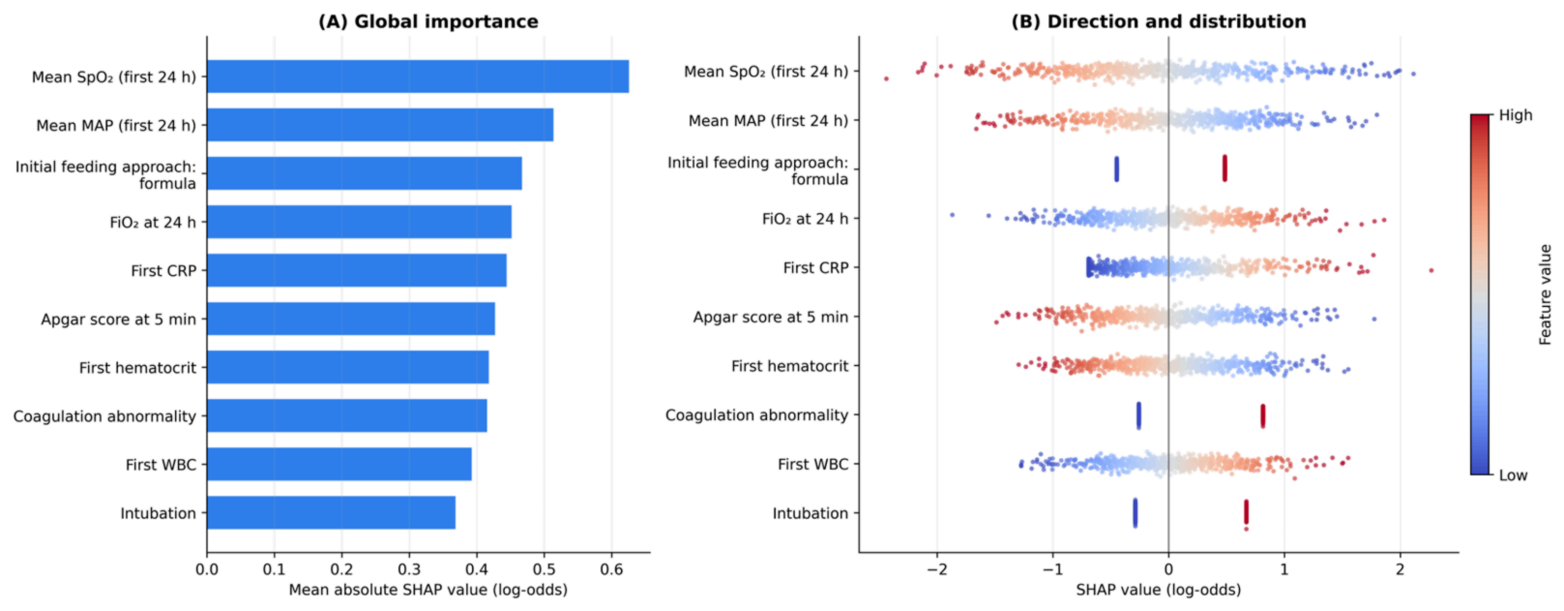
SHAP-based interpretation of the Step 3 logistic regression model in the held-out test set. (**A**) Mean absolute SHAP values for the 10 most influential predictors. (**B**) Distribution and direction of predictor contributions. Positive SHAP values indicate increased model output for BPD; colors represent low to high transformed feature values.

**Table 1:** Variables measured immediately after delivery (Birth characteristics).

Variable	Non-BPD (*n* = 999)	BPD (*n* = 1154)	*p*-Value
Sex, male	532 (53.3)	642 (55.6)	0.288
Airway malformations	15 (1.5)	38 (3.3)	0.011
HDP	84 (8.4)	152 (13.2)	<0.001
Maternal smoking	77 (7.7)	143 (12.4)	<0.001
Gestational age, weeks	29.41 ± 3.41	28.30 ± 3.41	<0.001
Birth weight, g	1310.04 ± 411.82	1152.36 ± 360.99	<0.001
Maternal age, years	27.52 ± 5.75	28.62 ± 6.01	<0.001
Apgar score at 1 min	6.08 ± 2.09	5.01 ± 2.01	<0.001
Apgar score at 5 min	8.01 ± 1.62	7.15 ± 1.79	<0.001

Data are presented as *n* (%) or mean ± standard deviation. Continuous variables were compared using Welch’s *t*-test; categorical variables were compared using the chi-square test or Fisher’s exact test, as appropriate. BPD, bronchopulmonary dysplasia; HDP: hypertensive disorders of pregnancy.

**Table 2:** Clinical and Laboratory Variables Within 24 Hours After Birth.

Variable	Non-BPD (*n* = 999)	BPD (*n* = 1154)	*p*-Value
Initial feeding approach within 24 h			<0.001
Breast milk	619 (62.0)	499 (43.2)	
Formula	380 (38.0)	655 (56.8)	
Neonatal RDS	615 (61.6)	864 (74.9)	<0.001
Respiratory failure	433 (43.3)	689 (59.7)	<0.001
Endotracheal intubation	118 (11.8)	320 (27.7)	<0.001
Mean SpO_2_ during the first 24 h, %	91.91 ± 2.91	89.23 ± 3.91	<0.001
FiO_2_ at 24 h	0.41 ± 0.12	0.47 ± 0.14	<0.001
Mean heart rate during the first 24 h, beats/min	155.33 ± 14.86	159.11 ± 13.90	<0.001
Mean arterial pressure during the first 24 h, mmHg	34.86 ± 5.67	32.04 ± 4.93	<0.001
Mean temperature during the first 24 h, °C	36.77 ± 0.39	36.59 ± 0.48	<0.001
First hemoglobin, g/dL	14.51 ± 1.76	13.68 ± 1.96	<0.001
First hematocrit, %	42.17 ± 4.86	40.03 ± 5.14	<0.001
First WBC count, ×10^3^/μL	10.15 ± 3.85	11.92 ± 4.52	<0.001
First platelet count, ×10^3^/μL	293.18 ± 93.08	252.40 ± 79.28	<0.001
First CRP, mg/L	1.60 ± 1.34	2.53 ± 2.00	<0.001
First AST, IU/L	35.74 ± 14.47	39.15 ± 19.17	<0.001
First ALT, IU/L	17.83 ± 8.25	20.52 ± 10.01	<0.001
Total parenteral nutrition within 24 h	535 (53.6)	692 (60.0)	0.003

The formula category included infants for whom formula feeding was attempted because breast-milk feeding could not be initiated; it did not necessarily indicate successful formula administration. SpO_2_, heart rate, mean arterial pressure, and temperature were summarized as means over the first 24 h; FiO_2_ was the value recorded at 24 h. For each laboratory variable, the first result available within 24 h after birth was used. Final blood-culture results were excluded from the prediction models. Data are presented as n (%) or mean ± standard deviation. RDS: respiratory distress syndrome; WBC: White blood cell count.

**Table 3:** Clinical Course Within the First 7 Days After Birth.

Variable	Non-BPD (*n* = 999)	BPD (*n* = 1154)	*p*-Value
IMV within 7 days	177 (17.7)	371 (32.1)	<0.001
Mean FiO_2_ through postnatal day 7	0.25 ± 0.09	0.30 ± 0.10	<0.001
Early-onset infection	29 (2.9)	51 (4.4)	0.082
Late-onset infection	214 (21.4)	305 (26.4)	0.008
Coagulation abnormality	181 (18.1)	403 (34.9)	<0.001
Thrombocytopenia	44 (4.4)	152 (13.2)	<0.001

Data are presented as n (%) or mean ± standard deviation. Continuous variables were compared using Welch’s *t*-test; categorical variables were compared using the chi-square test or Fisher’s exact test, as appropriate. First-week mean FiO_2_ was calculated as the equally weighted arithmetic mean of the seven daily mean FiO_2_ values. IMV: invasive mechanical ventilation; FiO_2_: fraction of inspired oxygen.

**Table 4:** Clinical characteristics and outcomes during hospitalization.

Variable	Non-BPD (*n* = 999)	BPD (*n* = 1154)	*p*-Value
PDA diagnosed by echocardiography after 24 h	403 (40.3)	589 (51.0)	<0.001
Final blood culture positive	44 (4.4)	135 (11.7)	<0.001
IMV	438 (43.8)	650 (56.3)	<0.001
Duration of IMV, days	11.22 ± 8.23	17.89 ± 10.34	<0.001
High-frequency ventilation	92 (9.2)	289 (25.0)	<0.001
Total days on supplemental oxygen	26.23 ± 17.33	37.62 ± 18.34	<0.001
Cumulative duration of antibiotic use, days	6.13 ± 3.17	7.76 ± 3.64	<0.001
Nutrition-support duration, days	15.31 ± 7.94	25.19 ± 9.78	<0.001
Length of hospital stay, days	50.46 ± 24.48	68.23 ± 25.05	<0.001
Transfusion	197 (19.7)	664 (57.5)	<0.001
Intravenous glucocorticoid therapy	15 (1.5)	225 (19.5)	<0.001
Caffeine therapy	587 (58.8)	853 (73.9)	<0.001
Death after BPD assessment	0 (0.0)	150 (13.0)	<0.001

Data are presented as n (%) or mean ± standard deviation. Continuous variables were compared using Welch’s *t*-test; categorical variables were compared using the chi-square test or Fisher’s exact test, as appropriate. BPD: bronchopulmonary dysplasia; IMV: invasive mechanical ventilation; PDA: Patent ductus arteriosus.

**Table 5:** Comparison of model performance across three feature sets reflecting different clinical time windows for predicting bronchopulmonary dysplasia.

Step	Model	CV Accuracy	CV AUROC	Test Accuracy	Test AUROC (95% CI)
Step 1	LR	0.675 (0.011)	0.734 (0.019)	0.687	0.751 (0.714–0.788)
	RF	0.646 (0.008)	0.714 (0.023)	0.669	0.724 (0.686–0.764)
	CatBoost	0.648 (0.020)	0.704 (0.023)	0.656	0.709 (0.670–0.749)
	XGBoost	0.627 (0.013)	0.685 (0.026)	0.638	0.685 (0.645–0.725)
	LightGBM	0.619 (0.006)	0.672 (0.024)	0.615	0.663 (0.622—0.705)
Step 2	LR	0.829 (0.022)	0.917 (0.016)	0.800	0.897 (0.873–0.919)
	RF	0.839 (0.019)	0.916 (0.020)	0.794	0.893 (0.868–0.917)
	CatBoost	0.845 (0.022)	0.924 (0.018)	0.788	0.892 (0.866–0.915)
	XGBoost	0.843 (0.028)	0.920 (0.018)	0.808	0.892 (0.866–0.915)
	LightGBM	0.836 (0.028)	0.918 (0.015)	0.808	0.895 (0.870–0.917)
Step 3	LR	0.849 (0.014)	0.928 (0.012)	0.839	0.923 (0.902–0.942)
	RF	0.847 (0.026)	0.926 (0.015)	0.820	0.910 (0.888–0.931)
	CatBoost	0.856 (0.019)	0.937 (0.012)	0.824	0.914 (0.891–0.934)
	XGBoost	0.857 (0.017)	0.933 (0.010)	0.822	0.910 (0.887–0.931)
	LightGBM	0.843 (0.023)	0.930 (0.014)	0.824	0.909 (0.886–0.929)

Cross-validation values are mean (standard deviation) across five stratified folds in the development set. Test AUROCs are presented with 95% percentile confidence intervals estimated using 2000 bootstrap resamples of the held-out test set. Predictor counts were 9, 26, and 32 for Steps 1, 2, and 3, respectively. AUROC, area under the receiver operating characteristic curve; CV, cross-validation; LR, logistic regression; RF, random forest.

## Data Availability

The datasets generated and/or analyzed during the current study are available from the corresponding author upon reasonable request due to privacy and ethical restrictions.

## Supplementary Materials

The following supporting information can be downloaded at: https://www.mdpi.com/article/10.3390/children13101299/s1, Table S1: Prespecified hyperparameter settings for the machine learning models; Table S2: Comparison of baseline characteristics between infants excluded because of missing predictor data and those included in the final analytic cohort; Table S3: Pairwise comparisons of test-set AUROC between logistic regression and tree-based algorithms across prediction windows; Table S4: Incremental discrimination between consecutive prediction windows; Table S5: Quantitative calibration and overall prediction error in the held-out test set; Table S6: Feature importance and mean absolute SHAP values for the Logistic regression model across Step 1, 2, and 3 feature sets; Table S7: Multivariable logistic regression analysis of clinically documented BPD using the Step 3 predictor set; Table S8: Multiple-imputation sensitivity analysis of discrimination, calibration, prediction error, and paired AUROC changes for logistic regression and random forest across prediction windows; Table S9: Predictor-specific missingness among infants excluded from the primary analysis and among all infants with ascertainable BPD status; Figure S1: Exploratory decision-curve analysis in the held-out test cohort; Figure S2: SHAP-based interpretation of the Step 1 logistic regression model in the held-out test set; Figure S3: SHAP-based interpretation of the Step 2 logistic regression model in the held-out test set.

## Abbreviations

The following abbreviations are used in this manuscript:

ALT	Alanine aminotransferase
AST	Aspartate aminotransferase
AUROC	Area under the receiver operating characteristic curve
BPD	Bronchopulmonary dysplasia
CRP	C-reactive protein
EMR	Electronic medical record
FiO_2_	Fraction of inspired oxygen
LightGBM	Light Gradient Boosting Machine
PDA	Patent ductus arteriosus
PMA	Postmenstrual age
SHAP	SHapley Additive exPlanations
SpO_2_	Peripheral oxygen saturation
XGBoost	Extreme gradient boosting
