# Supplementary material for: Sequential Prediction of Bronchopulmonary Dysplasia from Birth Through the First Week of Life Using Machine Learning
Source: Children (Basel). 2026 Sep 24;13(10):1299. doi: 10.3390/children13101299 (PMC13703402; doi:10.3390/children13101299)
Supplement: Supplementary file 1 [file children-13-01299-s001.zip › children-4528242-supplementary.pdf]

Supplementary Materials

Table S1. Prespecified hyperparameter settings for the machine-learning models.

Model	Hyperparameter settings
Logistic Regression	L2 penalty; C=0.7; lbfgs solver; maximum iterations=1,000
Random Forest	800 trees; maximum depth=10; minimum samples split=4; minimum leaf size=2
XGBoost	800 iterations; learning rate=0.03; maximum depth=6; subsample=0.8; column subsample=0.8; log-loss evaluation
LightGBM	800 iterations; learning rate=0.03; 31 leaves; subsample=0.8; column subsample=0.8
CatBoost	800 iterations; learning rate=0.03; depth=6; L2 leaf regularization=3
Common settings	Random seed=42; one stratified 70:30 development/test split; five-fold stratified cross-validation in the development set; preprocessing fitted within each training fold

All hyperparameters were prespecified and applied consistently across the feature sets. No feature-set-specific hyperparameter optimization was performed. Model performance was evaluated using five-fold stratified cross-validation within the training set, after which each model was refitted using the entire training set and evaluated once on the held-out test set.

Table S2. Comparison of baseline characteristics between infants excluded because of missing predictor data and those included in the final analytic cohort.

Characteristic	Excluded group (n = 463)	Available n	Final analytic cohort (n = 2,153)	p-value	Absolute SMD
BPD at 36 weeks' PMA	253 (54.6)	463	1,154 (53.6)	0.721	0.021
Sex, male	241 (52.2)	462	1,174 (54.5)	0.382	0.047
Airway malformations	9 (2.0)	459	53 (2.5)	0.638	0.034
Hypertensive disorders of pregnancy	53 (12.0)	443	236 (11.0)	0.598	0.031
Maternal smoking during pregnancy	33 (8.2)	403	220 (10.2)	0.245	0.07
Gestational age, weeks	27.34 ± 3.13	463	28.82 ± 3.46	<0.001	0.448
Birth weight, g	1,276.71 ± 358.88	461	1,225.52 ± 393.27	0.006	0.136
Maternal age, years	27.23 ± 4.18	442	28.11 ± 5.92	<0.001	0.172
Apgar score at 1 min	5.89 ± 1.97	428	5.50 ± 2.12	<0.001	0.189
Apgar score at 5 min	7.91 ± 1.50	426	7.55 ± 1.77	<0.001	0.221

Data are presented as mean ± standard deviation or n (%). Available n indicates the number of infants with nonmissing data for each characteristic in the excluded group. Percentages were calculated using the corresponding available sample size; the denominator for the final analytic cohort was 2,153. Continuous variables were compared using Welch's t-test, and categorical variables using the chi-square test. BPD, bronchopulmonary dysplasia; PMA, postmenstrual age; SMD, standardized mean difference.

Table S3. Pairwise comparisons of test-set AUROC between logistic regression and tree-based algorithms across prediction windows.

Step	Comparison	ΔAUROC (95% CI)	p-value	Holm-adjusted p-value
Step 1	LR − RF	0.0272 (0.0066 to 0.0478)	0.01	0.088
	LR − XGBoost	0.0665 (0.0369 to 0.0960)	<0.001	<0.001
	LR − LightGBM	0.0881 (0.0565 to 0.1197)	<0.001	<0.001
	LR − CatBoost	0.0430 (0.0179 to 0.0680)	<0.001	0.008
Step 2	LR − RF	0.0039 (−0.0116 to 0.0194)	0.626	1
	LR − XGBoost	0.0053 (−0.0084 to 0.0190)	0.447	1
	LR − LightGBM	0.0024 (−0.0131 to 0.0179)	0.762	1
	LR − CatBoost	0.0050 (−0.0079 to 0.0180)	0.444	1
Step 3	LR − RF	0.0126 (−0.0011 to 0.0264)	0.072	0.432
	LR − XGBoost	0.0123 (−0.0001 to 0.0247)	0.052	0.414
	LR − LightGBM	0.0135 (−0.0007 to 0.0277)	0.062	0.432
	LR − CatBoost	0.0090 (−0.0023 to 0.0202)	0.119	0.595

Differences are LR minus the comparator AUROC; positive values favor LR. Two-sided paired DeLong tests account for evaluation of both models in the same 646 test infants (346 with BPD and 300 without BPD). Confidence intervals are nominal 95% DeLong intervals and are not adjusted for multiplicity. Holm adjustment was applied across all 12 LR-versus-comparator tests. AUROC, area under the receiver operating characteristic curve; CI, confidence interval; LR, logistic regression; RF, random forest.

Table S4. Incremental discrimination between consecutive prediction windows.

Model	Comparison	ΔAUROC (95% CI)
Logistic regression	Step 2 minus Step 1	0.146 (0.110–0.181)
	Step 3 minus Step 2	0.026 (0.015–0.036)
Random forest	Step 2 minus Step 1	0.169 (0.133–0.206)
	Step 3 minus Step 2	0.017 (0.010–0.024)
XGBoost	Step 2 minus Step 1	0.207 (0.167–0.247)
	Step 3 minus Step 2	0.019 (0.010–0.028)
LightGBM	Step 2 minus Step 1	0.231 (0.190–0.272)
	Step 3 minus Step 2	0.014 (0.005–0.025)
CatBoost	Step 2 minus Step 1	0.184 (0.144–0.222)
	Step 3 minus Step 2	0.022 (0.013–0.030)

Differences are newer minus older AUROC. In each of 2,000 bootstrap resamples, the same infant indices were used for both prediction windows. Intervals are nominal 95% percentile intervals, without multiplicity adjustment; these revision-stage comparisons are exploratory. No bootstrap tail-count p-values are reported. The comparisons evaluate discrimination within the selected cohort, not the clinical benefit of waiting until a later prediction time.

Table S5. Quantitative calibration and overall prediction error in the held-out test set.

Step	Model	Calibration-in-the-large (95% CI)	Calibration slope (95% CI)	Brier score (95% CI)
Step 1	Logistic regression	0.058 (-0.111–0.228)	1.085 (0.869–1.300)	0.202 (0.190–0.215)
	Random forest	0.028 (-0.142–0.197)	0.941 (0.738–1.144)	0.211 (0.198–0.224)
	XGBoost	0.030 (-0.166–0.225)	0.389 (0.292–0.485)	0.245 (0.224–0.266)
	LightGBM	0.054 (-0.154–0.262)	0.276 (0.201–0.351)	0.270 (0.246–0.293)
	CatBoost	0.080 (-0.103–0.263)	0.545 (0.418–0.671)	0.224 (0.206–0.242)
Step 2	Logistic regression	0.109 (-0.115–0.333)	0.890 (0.754–1.025)	0.130 (0.114–0.147)
	Random forest	0.082 (-0.095–0.259)	1.904 (1.618–2.190)	0.144 (0.133–0.155)
	XGBoost	0.248 (-0.027–0.523)	0.533 (0.454–0.613)	0.144 (0.124–0.166)
	LightGBM	0.316 (-0.030–0.663)	0.338 (0.288–0.388)	0.154 (0.131–0.179)
	CatBoost	0.209 (-0.042–0.461)	0.651 (0.553–0.749)	0.140 (0.122–0.160)
Step 3	Logistic regression	0.009 (-0.227–0.246)	0.948 (0.804–1.092)	0.112 (0.097–0.128)
	Random forest	0.075 (-0.100–0.250)	2.262 (1.924–2.600)	0.139 (0.129–0.149)
	XGBoost	0.170 (-0.112–0.451)	0.586 (0.500–0.673)	0.128 (0.109–0.149)
	LightGBM	0.172 (-0.184–0.527)	0.354 (0.302–0.406)	0.141 (0.119–0.165)
	CatBoost	0.123 (-0.136–0.382)	0.718 (0.611–0.825)	0.122 (0.104–0.141)

Calibration-in-the-large is the intercept from logistic regression with the logit of predicted risk as an offset (slope fixed at 1). Calibration slope is estimated in a separate logistic regression with a freely estimated intercept. Calibration intervals are model-based Wald 95% intervals. Predicted probabilities are clipped to $[10^{-6}, 1\text{-}10^{-6}]$ only for calibration fitting. Ideal calibration-in-the-large and slope are 0 and 1. Brier score is mean squared prediction error; lower is better. Brier intervals use 2,000 test-set bootstrap resamples. Calibration models are diagnostic only; predictions were not recalibrated.

Table S6. Feature importance and mean absolute SHAP values for the Logistic Regression model across Step 1, 2, and 3 feature sets.

Feature sets		Step 1		Step 2		Step 3	
		Absolute coefficient	Mean ∣SHAP∣	Absolute coefficient	Mean ∣SHAP∣	Absolute coefficient	Mean ∣SHAP∣
Immediately after delivery	Male sex	0.115	0.057	0.098	0.049	0.086	0.043
	Airway malformations	0.060	0.013	0.108	0.023	0.138	0.029
	HDP	0.144	0.068	0.147	0.069	0.133	0.063
	Maternal smoking	0.143	0.092	0.183	0.117	0.174	0.112
	Gestational age	0.298	0.252	0.291	0.246	0.318	0.269
	Birth weight	0.355	0.271	0.277	0.211	0.231	0.176
	Maternal age	0.207	0.169	0.209	0.171	0.203	0.166
	Apgar score at 1 min	0.516	0.393	0.472	0.360	0.454	0.346
	Apgar score at 5 min	0.533	0.414	0.568	0.441	0.550	0.427
Within 24 hours after birth	Initial feeding approach: formula			0.924	0.462	0.934	0.467
	Neonatal RDS			0.266	0.244	0.242	0.222
	Respiratory failure			0.286	0.286	0.329	0.329
	Intubation			0.378	0.363	0.384	0.368
	Mean SpO_2 (first 24 h)			0.829	0.634	0.818	0.625
	FiO_2 at 24 h			0.530	0.431	0.556	0.452
	Mean heart rate (first 24 h)			0.331	0.262	0.317	0.251
	Mean MAP (first 24 h)			0.564	0.480	0.603	0.514
	Mean temperature (first 24 h)			0.392	0.311	0.344	0.273
	First hemoglobin			0.398	0.318	0.418	0.334
	First hematocrit			0.457	0.366	0.523	0.418
	First WBC			0.495	0.394	0.493	0.392
	First platelet count			0.481	0.386	0.443	0.355
	First CRP			0.561	0.451	0.552	0.444
	First AST			0.269	0.222	0.203	0.168
	First ALT			0.272	0.212	0.276	0.215
	TPN within 24 h			0.161	0.155	0.170	0.164
First 7 days after birth	IMV within 7 days					0.283	0.258
	Mean FiO_2 through day 7					0.433	0.348
	Early-onset infection					0.017	0.010
	Late-onset infection					0.157	0.128
	Coagulation abnormality					0.474	0.415
	Thrombocytopenia					0.292	0.142

Steps 1–3 represent cumulative predictors available immediately after the initial postnatal assessment, within 24 hours, and through day 7, respectively. Blank cells indicate predictors not included. Absolute coefficients reflect numeric predictor standardization and categorical dummy coding. Mean ∣SHAP∣ values were calculated in the held-out test set on the log-odds scale. The formula category included attempted feeding and did not necessarily indicate actual formula administration. ALT, alanine aminotransferase; AST, aspartate

aminotransferase; CRP, C-reactive protein; FiO_2, fraction of inspired oxygen; HDP, hypertensive disorders of pregnancy; IMV, invasive mechanical ventilation; MAP, mean arterial pressure; RDS, respiratory distress syndrome; SHAP, SHapley Additive exPlanations; SpO_2, peripheral oxygen saturation; TPN, total parenteral nutrition; WBC, white blood cell count.

Table S7. Multivariable logistic regression analysis of clinically documented BPD using the Step 3 predictor set.

	Predictor	Coefficient	Standard error	OR (95% CI)	p-value
Immediately after delivery	Male sex	0.089	0.138	1.093 (0.835-1.432)	0.516
	Airway malformations	1.276	0.482	3.582 (1.392-9.221)	0.008
	HDP	0.390	0.229	1.477 (0.944-2.312)	0.088
	Maternal smoking	0.657	0.223	1.928 (1.246-2.983)	0.003
	Gestational age	-0.099	0.020	0.906 (0.870-0.942)	<0.001
	Birth weight, per 100-g increase	-0.080	0.018	0.923 (0.892-0.956)	<0.001
	Maternal age	0.027	0.011	1.028 (1.005-1.051)	0.017
	Apgar score at 1 min	-0.212	0.033	0.809 (0.758-0.863)	<0.001
	Apgar score at 5 min	-0.281	0.041	0.755 (0.697-0.818)	<0.001
Within 24 hours after birth	Initial feeding approach: formula	0.845	0.138	2.327 (1.775-3.052)	<0.001
	Neonatal RDS	0.531	0.148	1.701 (1.272-2.275)	<0.001
	Respiratory failure	0.575	0.138	1.778 (1.357-2.329)	<0.001
	Intubation	0.923	0.182	2.516 (1.762-3.592)	<0.001
	Mean SpO_2 (first 24 h)	-0.203	0.021	0.816 (0.784-0.850)	<0.001
	FiO_2 at 24 h, per 0.10 increase	0.035	0.005	1.036 (1.025-1.047)	<0.001
	Mean heart rate (first 24 h)	0.020	0.005	1.020 (1.011-1.030)	<0.001
	Mean MAP (first 24 h)	-0.113	0.013	0.893 (0.870-0.917)	<0.001
	Mean temperature (first 24 h)	-0.915	0.163	0.400 (0.291-0.551)	<0.001
	First hemoglobin	-0.225	0.037	0.798 (0.742-0.859)	<0.001
	First hematocrit	-0.108	0.014	0.897 (0.873-0.922)	<0.001
	First WBC, per 1,000 cells/μL increase	0.113	0.017	1.119 (1.083-1.157)	<0.001
	First platelet count, per 50,000 cells/μL increase	-0.282	0.040	0.754 (0.698-0.816)	<0.001
	First CRP	0.330	0.043	1.391 (1.280-1.512)	<0.001
	First AST	0.013	0.004	1.013 (1.005-1.021)	0.002
	First ALT	0.029	0.008	1.030 (1.014-1.045)	<0.001
	TPN within 24 h	0.375	0.139	1.455 (1.107-1.911)	0.007
First 7 days after birth	IMV within 7 days	0.802	0.162	2.229 (1.623-3.062)	<0.001
	Mean FiO_2 through day 7, per 0.10 increase	0.485	0.072	1.625 (1.412-1.869)	<0.001
	Early-onset infection	0.245	0.354	1.278 (0.638-2.558)	0.489
	Late-onset infection	0.351	0.158	1.421 (1.043-1.936)	0.026
	Coagulation abnormality	1.117	0.156	3.056 (2.251-4.148)	<0.001
	Thrombocytopenia	1.169	0.254	3.218 (1.955-5.297)	<0.001

Odds ratios are adjusted for all other predictors in the model and expressed per indicated increment for continuous variables.

Table S8. Multiple-imputation sensitivity analysis of discrimination, calibration and prediction error for logistic regression and random forest across prediction windows.

Model	Step	Analysis / test population	Test n	BPD n	AUROC	95% CI lower	95% CI upper	AUPRC	Brier score	CITL	Calibration slope
LR	1	Complete case / original test	646	346	0.751	0.714	0.787	0.777	0.202	0.058	1.085
RF	1	Complete case / original test	646	346	0.724	0.686	0.762	0.748	0.211	0.028	0.941
LR	1	MI / expanded test	785	422	0.747	0.715	0.780	0.772	0.204	0.037	1.043
RF	1	MI / expanded test	785	422	0.729	0.694	0.764	0.742	0.210	0.031	0.962
LR	2	Complete case / original test	646	346	0.897	0.873	0.920	0.913	0.130	0.109	0.890
RF	2	Complete case / original test	646	346	0.893	0.869	0.916	0.907	0.144	0.082	1.904
LR	2	MI / expanded test	785	422	0.893	0.870	0.913	0.911	0.134	0.094	0.903
RF	2	MI / expanded test	785	422	0.893	0.872	0.915	0.907	0.143	0.064	1.923
LR	3	Complete case / original test	646	346	0.923	0.903	0.941	0.935	0.112	0.009	0.948
RF	3	Complete case / original test	646	346	0.910	0.888	0.930	0.923	0.139	0.075	2.262
LR	3	MI / expanded test	785	422	0.913	0.892	0.931	0.926	0.120	0.006	0.937
RF	3	MI / expanded test	785	422	0.902	0.882	0.922	0.916	0.142	0.064	2.183

MI results were based on predicted probabilities averaged across 20 imputations. AUROC 95% CIs were estimated using 2,000 test-set bootstrap resamples. The original and expanded test cohorts differed in composition. Ideal CITL and calibration slope values are 0 and 1, respectively; lower Brier scores indicate better performance. LR, logistic regression; RF, random forest; MI, multiple imputation; BPD, bronchopulmonary dysplasia; AUROC, area under the receiver operating characteristic curve; AUPRC, area under the precision–recall curve; CI, confidence interval; CITL, calibration-in-the-large.

Table S9. Predictor-specific missingness among infants excluded from the primary analysis and among all infants with ascertainable BPD status.

Predictor	Step	Missing (*n*)	Excluded missing (%)	Combined missing(%)
Sex, male	1	1	0.002	0.000
Airway malformations		7	0.015	0.003
HDP		0	0.000	0.000
Maternal smoking		1	0.002	0.000
Gestational age, weeks		0	0.000	0.000
Birth weight, g		2	0.004	0.001
Maternal age, years		51	0.110	0.019
Apgar score at 1 min		35	0.076	0.013
Apgar score at 5 min		37	0.080	0.014
Initial feeding approach within 24 h	2	8	0.017	0.003
Neonatal RDS		8	0.017	0.003
Respiratory failure		4	0.009	0.002
Endotracheal intubation		5	0.011	0.002
Mean SpO_2 during the first 24 h, %		52	0.112	0.020
FiO_2 at 24 h		57	0.123	0.022
Mean heart rate during the first 24 h, beats/min		55	0.119	0.034
Mean arterial pressure during the first 24 h, mmHg		67	0.145	0.029
Mean temperature during the first 24 h, °C		63	0.136	0.033
First hemoglobin, g/dL		82	0.177	0.031
First hematocrit, %		84	0.181	0.032
First WBC count, $\times 10^3$/μL		87	0.188	0.033
First platelet count, $\times 10^3$/μL		82	0.177	0.031
First CRP, mg/L		63	0.136	0.024
First AST, IU/L		83	0.179	0.032
First ALT, IU/L		85	0.184	0.032
Total parenteral nutrition within 24 h		26	0.056	0.010
IMV within 7 days	3	20	0.043	0.008
Mean FiO_2 through postnatal day 7		65	0.140	0.025
Early-onset infection		21	0.045	0.008
Late-onset infection		17	0.037	0.006
Coagulation abnormality		28	0.060	0.011
Thrombocytopenia		48	0.104	0.018

Values represent missing predictor data before imputation. Percentages were calculated using 463 infants excluded from the primary complete-case analysis and all 2616 infants with ascertainable BPD status as the respective denominators. An infant could have missing values for multiple predictors. BPD, bronchopulmonary dysplasia.

Supplementary Figure Legends

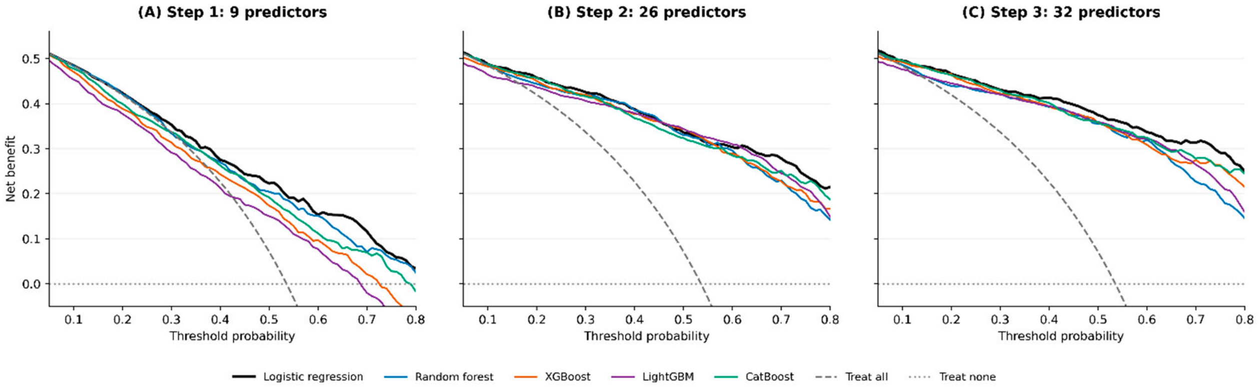

Figure S1. Exploratory decision-curve analysis in the held-out test cohort. Panels show the five algorithms using Step 1 (A), Step 2 (B), and Step 3 (C) predictors. Net benefit was evaluated at threshold probabilities from 0.05 to 0.80 and compared with hypothetical treat-all and treat-none strategies. Curves show point estimates; values below −0.05 are outside the displayed range. The threshold range is exploratory and does not indicate clinically recommended cutoffs or demonstrated treatment benefit.

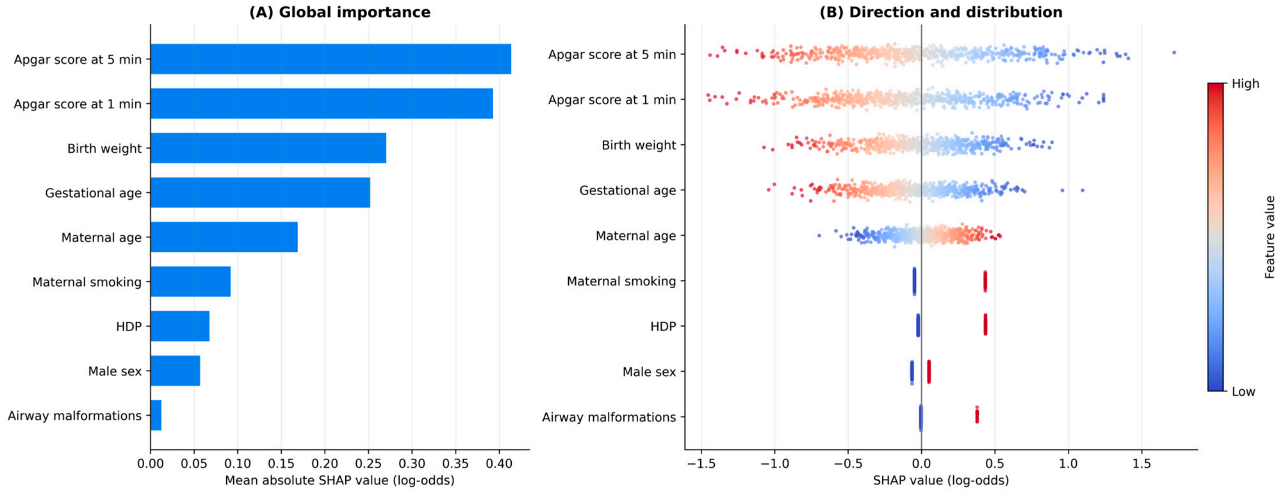

Figure S2. SHAP-based interpretation of the Step 1 logistic regression model in the held-out test set. (A) Mean absolute SHAP values for all nine predictors. (B) Distribution and direction of predictor contributions. Positive SHAP values increase the model output for clinically documented BPD. Values are expressed on the log-odds scale. Colors indicate low to high transformed feature values.

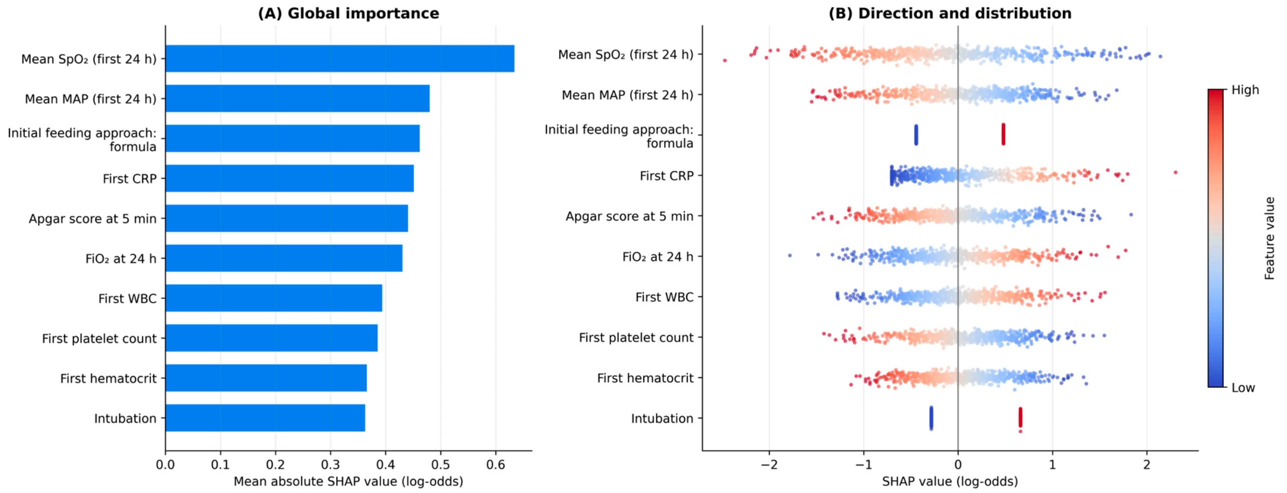

Figure S3. SHAP-based interpretation of the Step 2 logistic regression model in the held-out test set. (A) Mean absolute SHAP values for the 10 most influential predictors. (B) Distribution and direction of predictor contributions. Positive SHAP values increase the model output for clinically documented BPD. Values are expressed on the log-odds scale. Colors indicate low to high transformed feature values. Initial feeding approach: formula is an indicator relative to breast milk. This category includes attempted formula feeding and does not necessarily indicate actual formula administration.
